# Complete genome sequence of *Rhizobium leguminosarum* bv. viciae SRDI969, an acid-tolerant, efficient N_2_-fixing microsymbiont of *Vicia faba*

**DOI:** 10.1128/MRA.00489-23

**Published:** 2023-08-01

**Authors:** MacLean G. Kohlmeier, Elizabeth A. Farquharson, Ross A. Ballard, Graham W. O’Hara, Jason J. Terpolilli

**Affiliations:** 1 Legume Rhizobium Sciences, Food Futures Institute, Murdoch University, Perth, Western Australia, Australia; 2 South Australian Research and Development Institute, Urrbrae, South Australia, Australia; 3 University of Adelaide, School of Agriculture, Food and Wine, Adelaide, South Australia, Australia; University of Arizona, Tucson, Arizona, USA

**Keywords:** genomics, symbiosis, nitrogen fixation, DNA sequencing

## Abstract

We report the complete genome sequence of *Rhizobium leguminosarum* bv. viciae SRDI969, an acid-tolerant, efficient nitrogen-fixing microorganism of *Vicia faba*. The 6.8 Mbp genome consists of a chromosome and four plasmids, with the symbiosis and nitrogen fixation genes encoded on the chromosome.

## ANNOUNCEMENT

*Vicia faba* L. (faba bean) is a grain legume grown on 2.5 Mha globally (1) that forms a nitrogen-fixing symbiosis with *Rhizobium* bacteria. In Australia, faba bean is cultivated on 260,000 ha and routinely inoculated with an elite strain of rhizobia (2). However, rhizobia that nodulate faba beans are typically acid-sensitive, restricting expansion of the grain into areas containing acid soils. A new inoculant strain, *Rhizobium leguminosarum* bv. viciae SRDI969, has been selected for commercial release in late 2023 to provide improved nodulation and nitrogen fixation of faba bean on acid soils, compared with the current Australian commercial inoculant *Rhizobium laguerrae* WSM1455.

SRDI969 was isolated on 20 January 2015 on YMA-CR (3) from an uninoculated field pea (*Pisum sativum* L. cv. Kaspa) root nodule collected in August 2014 near Riverton, South Australia, by the South Australian Research and Development Institute (SARDI). A frozen stock was streaked onto ½LA agar (4) and incubated at 28°C until single colonies were observed. A single colony was transferred to ½LA broth and grown overnight on an orbital shaker at 250 rpm at 28°C.

Genomic DNA for short-read sequencing was isolated with a Qiagen DNeasy Blood and Tissue Kit using standard procedures. The library was prepared using a Nextera XT DNA Library Preparation Kit and sequenced by an Illumina NextSeq 500 platform with 2 × 150 bp chemistry, generating a total of 2,859,751 paired end reads. Genomic DNA for long-read sequencing was isolated using a phenol-chloroform extraction protocol as previously described (5). The library was prepared with an Oxford Nanopore Technologies Rapid Sequencing Kit SQK-RAD004 and sequenced by a MinION Mk1B device with a R9.4.1 flowcell, generating 2,732,148,120 bp of sequence across 402,143 reads with an *N*_50_ value of 10,806 bp. Reads were basecalled with Guppy v 4.2.2 using the high accuracy model and the config file “dna_r9.4.1_450bps_hac.cfg.” Reads below 10 kbp and quality score 10 were removed with NanoFilt v 2.7.1 (6).

The assembly generated using Flye v 2.8.3 (7) consists of 6,842,488 bp across five circular replicons, which were polished five times with unfiltered nanopore reads using Racon v 1.4.17 (8) and six times with Illumina reads using Pilon v 1.23 (9). The assembled genome was annotated with NCBI Prokaryotic Genome Annotation Pipeline (PGAP) v 6.2 (10), with 6,657 predicted genes composed of 6,390 coding sequences, three complete sets of rRNAs (5S, 16S, and 23S rRNAs) plus one additional 5S rRNA gene copy, 52 tRNAs, and four non-coding RNAs. Default parameters were used for all software unless otherwise specified.

**Fig 1:**
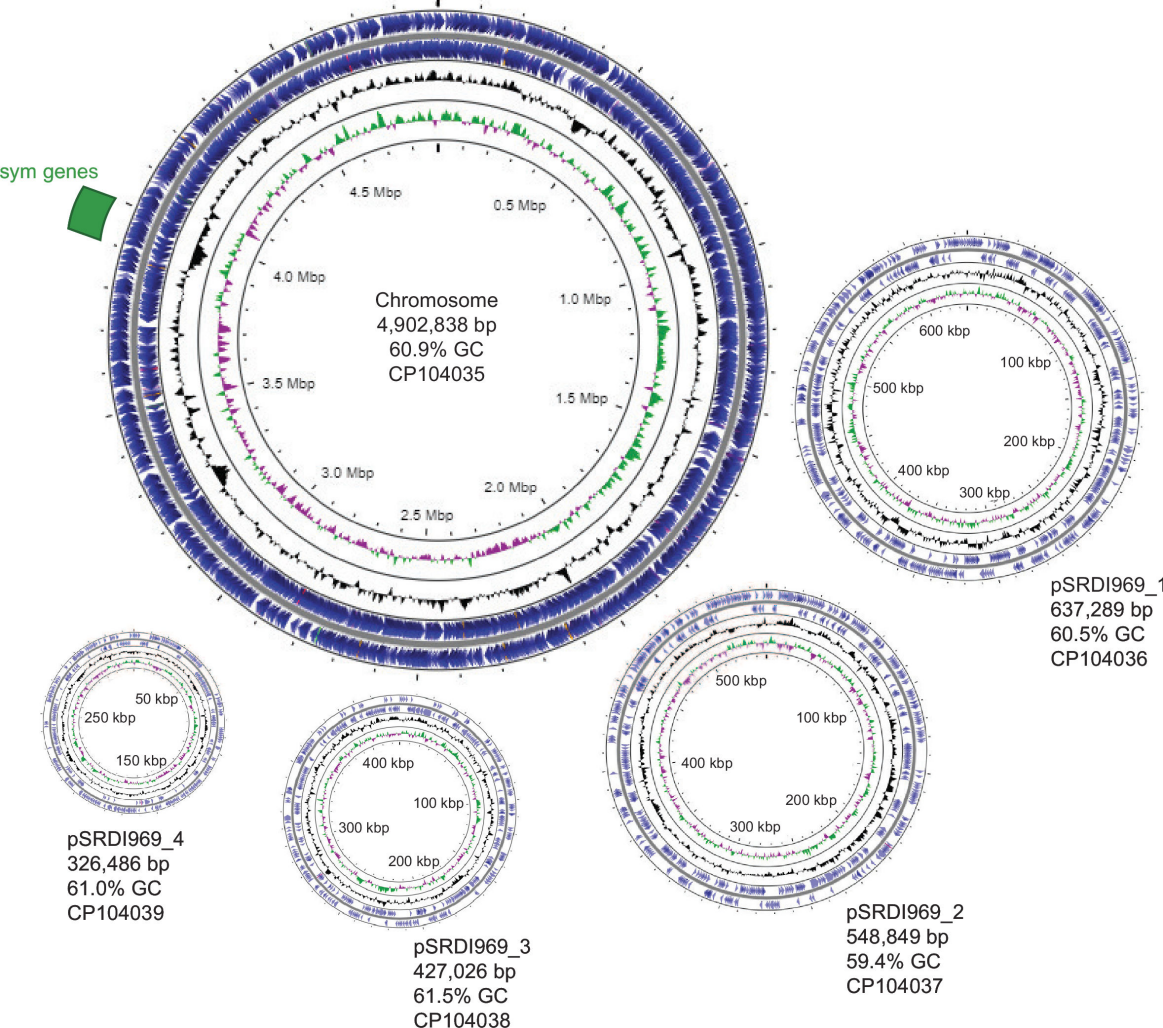
Genome architecture of *Rhizobium leguminosarum* bv. viciae SRDI969. The plasmids are shown at the same relative scale, and the chromosome is at one-fourth of that scale. Inner circles indicate deviations in GC skew (green/purple) and GC content (black). Coding sequences are shown in blue, and the symbiosis island is marked in green. Figures were made using Proksee (13) and modified in Adobe Illustrator 2023.

The final assembly includes a 4.9 Mbp chromosome and four plasmids designated pSRDI969_1 through 4 with sizes of 0.64, 0.55, 0.43, and 0.33 Mbp, respectively (Fig. 1). The symbiosis genes (*nod*, *nif*, and *fix*) are located within a chromosomally encoded ~75 kbp symbiosis island, which is unusual as symbiosis genes are frequently found on plasmids in *Rhizobium* spp. (11). The symbiosis genes are similar in structure and organization to the well-studied *R. leguminosarum* bv. viciae strain 3841 (12).

## Data Availability

This genome project is indexed at GenBank under BioProject accession number PRJNA783123. The complete sequences for the chromosomes, pSRDI969_1, pSRDI969_2, pSRDI969_3, and pSRDI969_4 can be found at GenBank under accession numbers CP104035, CP104036, CP104037, CP104038, and CP104039, respectively. Basecalled ONT reads can be found at SRA accession number SRX20543113, and raw ONT and Illumina reads can be found at SRA accession numbers SRX20520581 and SRX20520580, respectively.
